# (2*E*)-1-(4,4′′-Difluoro-5′-meth­oxy-1,1′:3′,1′′-terphenyl-4′-yl)-3-(2,6-difluoro­phen­yl)prop-2-en-1-one

**DOI:** 10.1107/S160053681201820X

**Published:** 2012-04-28

**Authors:** Hoong-Kun Fun, Tze Shyang Chia, S. Samshuddin, B. Narayana, B. K. Sarojini

**Affiliations:** a X-ray Crystallography Unit, School of Physics, Universiti Sains Malaysia, 11800 USM, Penang, Malaysia; b Department of Studies in Chemistry, Mangalore University, Mangalagangotri 574 199, India; c Department of Chemistry, P. A. College of Engineering, Nadupadavu, Mangalore 574 153, India

## Abstract

In the title compound, C_28_H_18_F_4_O_2_, the central benzene ring makes dihedral angles of 44.27 (6), 56.33 (5) and 77.27 (6)° with the two adjacent fluoro­benzene rings and terminal difluoro-substituted benzene ring, respectively. The dihedral angle between the fluoro­benzene rings is 87.81 (6)°. The meth­oxy and prop-2-en-1-one groups are essentially coplanar with their attached benzene rings, as indicated by their C—O—C_ar_—C_ar_ [−0.06 (15)°] and C—C—C_ar_—C_ar_ [4.5 (2)°] (ar = aromatic) torsion angles. In the crystal, mol­ecules are linked by C—H⋯F and C—H⋯O hydrogen bonds into sheets lying parallel to the *ac* plane. The crystal structure also features C—H⋯π inter­actions.

## Related literature

For related structures and background to terphenyl chalcones, see: Fun *et al.* (2011 ▶, 2012 ▶). For the stability of the temperature controller used in the data collection, see: Cosier & Glazer (1986 ▶). For bond-length data, see: Allen *et al.* (1987 ▶).
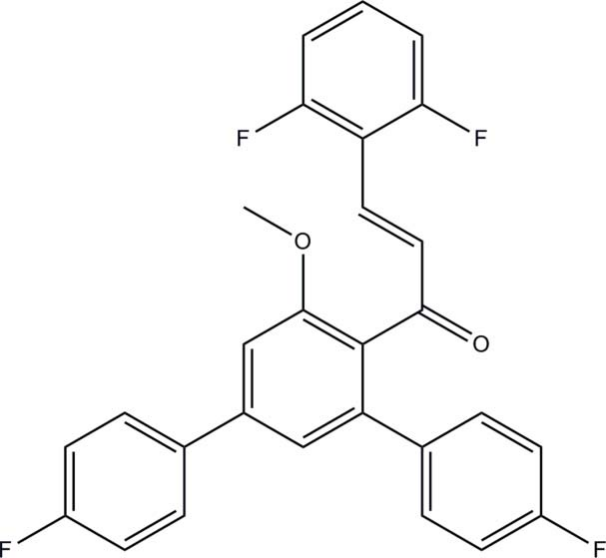


## Experimental

### 

#### Crystal data

C_28_H_18_F_4_O_2_
*M* *_r_* = 462.42
Triclinic, 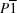

*a* = 8.9624 (5) Å
*b* = 10.2127 (6) Å
*c* = 13.3281 (7) Å
α = 67.780 (1)°
β = 86.776 (1)°
γ = 85.293 (1)°
*V* = 1125.10 (11) Å^3^
*Z* = 2
Mo *K*α radiation
μ = 0.11 mm^−1^
*T* = 100 K
0.25 × 0.20 × 0.11 mm

#### Data collection

Bruker APEX Duo CCD diffractometer
Absorption correction: multi-scan (*SADABS*; Bruker, 2009 ▶) *T*_min_ = 0.974, *T*_max_ = 0.989
28220 measured reflections
8063 independent reflections
6097 reflections with *I* > 2σ(*I*)
*R*_int_ = 0.035

#### Refinement

*R*[*F* ^2^ > 2σ(*F* ^2^)] = 0.046
*wR*(*F* ^2^) = 0.144
*S* = 1.02
8063 reflections
308 parameters
H-atom parameters constrained
Δρ_max_ = 0.40 e Å^−3^
Δρ_min_ = −0.35 e Å^−3^

### 

Data collection: *APEX2* (Bruker, 2009 ▶); cell refinement: *SAINT* (Bruker, 2009 ▶); data reduction: *SAINT*; program(s) used to solve structure: *SHELXTL* (Sheldrick, 2008 ▶); program(s) used to refine structure: *SHELXTL*; molecular graphics: *SHELXTL*; software used to prepare material for publication: *SHELXTL* and *PLATON* (Spek, 2009 ▶).

## Supplementary Material

Crystal structure: contains datablock(s) global, I. DOI: 10.1107/S160053681201820X/hb6753sup1.cif

Structure factors: contains datablock(s) I. DOI: 10.1107/S160053681201820X/hb6753Isup2.hkl

Supplementary material file. DOI: 10.1107/S160053681201820X/hb6753Isup3.cml

Additional supplementary materials:  crystallographic information; 3D view; checkCIF report

## Figures and Tables

**Table 1:** Hydrogen-bond geometry (Å, °) *Cg*1 and *Cg*2 are the centroids of the C1–C6 and C7–C12 rings, respectively.

*D*—H⋯*A*	*D*—H	H⋯*A*	*D*⋯*A*	*D*—H⋯*A*
C2—H2*A*⋯F3^i^	0.93	2.46	3.3655 (18)	164
C8—H8*A*⋯F4^ii^	0.93	2.45	3.3726 (13)	170
C24—H24*A*⋯O2^iii^	0.93	2.57	3.4371 (14)	155
C20—H20*A*⋯*Cg*1^iv^	0.93	2.83	3.5082 (14)	130
C27—H27*A*⋯*Cg*2^v^	0.93	2.68	3.4068 (12)	136
C28—H28*B*⋯*Cg*2^vi^	0.96	2.90	3.7990 (15)	157

Symmetry codes: (i) 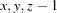; (ii) 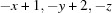; (iii) 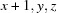; (iv) 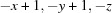; (v) 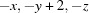; (vi) 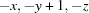.

**Fig. 1.**
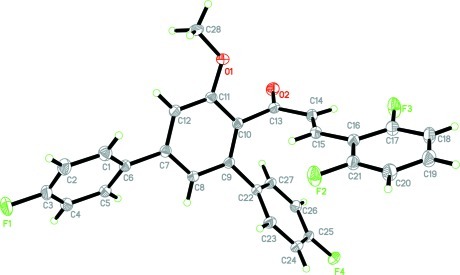
The molecular structure of the title compound with 50% probability displacement ellipsoids.

**Fig. 2.**
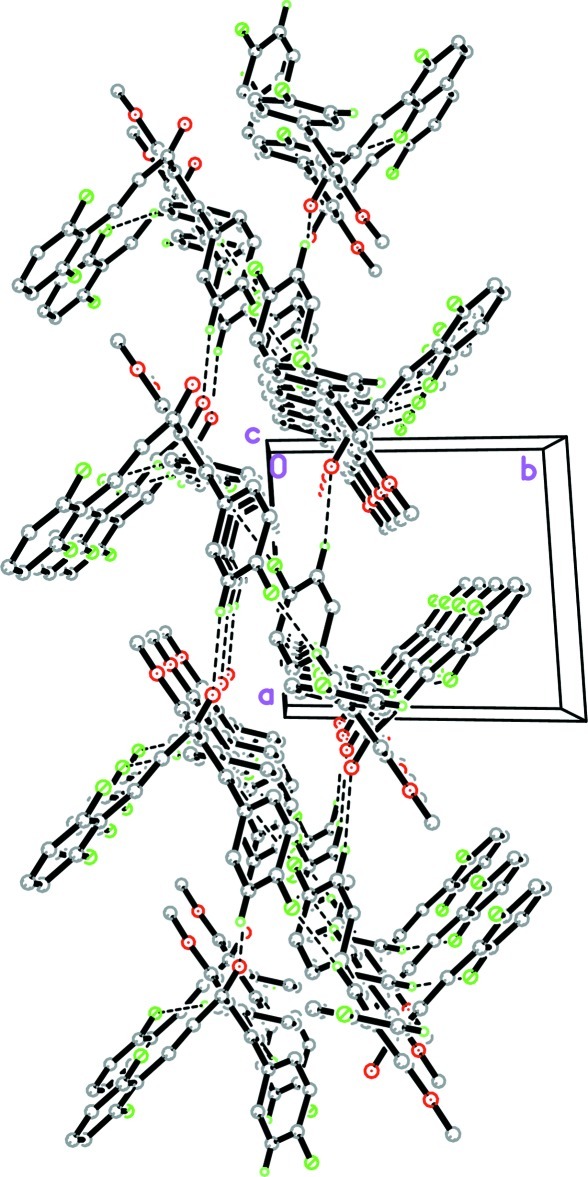
The crystal packing of the title compound. The dashed lines represent the hydrogen bonds.

## supplementary crystallographic information

### Comment

In continuation of our work on synthesis of terphenyl chalcones (Fun *et al.*, 2011), the title compound is prepared and its crystal structure is reported. The starting material of the title compound was prepared from 4,4'-difluoro chalcone by several steps (Fun *et al.*, 2012).

In the title compound (Fig. 1), the central benzene ring (C7–C12) makes dihedral angles of 44.27 (6), 56.33 (5) and 77.27 (6)° with the two adjacent fluoro-substituted benzene rings (C1–C6 & C22–C27) and terminal difluoro-substituted benzene ring (C16–C21), respectively. The dihedral angle between the fluoro-substituted benzene rings is 87.81 (6)°. The methoxy (O1/C28) and prop-2-en-1-one (O2/C13–C15) groups are essentially coplanar with C7–C12 and C16–C21 rings, respectively as indicated by their torsion angles C28—O1—C11—C12 = -0.06 (15)° and C14—C15—C16—C17 = 4.5 (2)°. Bond lengths and angles are comparable to those in related structures (Fun *et al.*, 2011,, 2012).

In the crystal (Fig. 2), molecules are linked by C2—H2A···F3, C8—H8A···F4 and C24—H24A···O2 hydrogen bonds (Table 1) into two dimensional networks parallel to *ac* plane. The crystal also features C—H···π interactions (Table 1), involving *Cg*1 and *Cg*2 which are the centroids of C1—C6 and C7—C12 rings, respectively.

### Experimental

To a mixture of

1-(4,4''-difluoro-5'-methoxy-1,1':3',1''-terphenyl-4'-yl)ethanone (0.338 g, 0.001 mol) and 2,6-difluorobenzaldehyde (0.142 g, 0.001 mol) in 30 ml e thanol, 0.5 ml of 10% sodium hydroxide solution was added and stirred at 5–10 °C for 3 h. The precipitate formed was collected by filtration and then purified by recrystallization from ethanol. Colourless blocks were grown from acetone solution by slow evaporation and the yield of the compound was 72% (m.p.: 405 K).

### Refinement

All H atoms were positioned geometrically [C—H = 0.93 and 0.96 Å] and refined using a riding model with *U*_iso_(H) = 1.2 or 1.5*U*_eq_(C). A rotating group model was applied to the methyl group. Three outliers (4 - 5 1), (0 - 3 2) and (3 - 5 2) were omitted.

### Crystal data

**Table:**

C_28_H_18_F_4_O_2_	*Z* = 2
*M**_r_* = 462.42	*F*(000) = 476
Triclinic, *P*1	*D*_x_ = 1.365 Mg m^−^^3^
Hall symbol: -P 1	Mo *K*α radiation, λ = 0.71073 Å
*a* = 8.9624 (5) Å	Cell parameters from 7563 reflections
*b* = 10.2127 (6) Å	θ = 2.3–32.4°
*c* = 13.3281 (7) Å	µ = 0.11 mm^−^^1^
α = 67.780 (1)°	*T* = 100 K
β = 86.776 (1)°	Block, colourless
γ = 85.293 (1)°	0.25 × 0.20 × 0.11 mm
*V* = 1125.10 (11) Å^3^	

### Data collection

**Table:**

Bruker APEX Duo CCD diffractometer	8063 independent reflections
Radiation source: fine-focus sealed tube	6097 reflections with *I* > 2σ(*I*)
Graphite monochromator	*R*_int_ = 0.035
φ and ω scans	θ_max_ = 32.5°, θ_min_ = 1.7°
Absorption correction: multi-scan (*SADABS*; Bruker, 2009)	*h* = −13→13
*T*_min_ = 0.974, *T*_max_ = 0.989	*k* = −15→14
28220 measured reflections	*l* = −20→18

### Refinement

**Table:**

Refinement on *F*^2^	Primary atom site location: structure-invariant direct methods
Least-squares matrix: full	Secondary atom site location: difference Fourier map
*R*[*F*^2^ > 2σ(*F*^2^)] = 0.046	Hydrogen site location: inferred from neighbouring sites
*wR*(*F*^2^) = 0.144	H-atom parameters constrained
*S* = 1.02	*w* = 1/[σ^2^(*F*_o_^2^) + (0.0774*P*)^2^ + 0.2553*P*] where *P* = (*F*_o_^2^ + 2*F*_c_^2^)/3
8063 reflections	(Δ/σ)_max_ < 0.001
308 parameters	Δρ_max_ = 0.40 e Å^−^^3^
0 restraints	Δρ_min_ = −0.35 e Å^−^^3^

### Special details

**Table:**

Experimental. The crystal was placed in the cold stream of an Oxford Cryosystems Cobra open-flow nitrogen cryostat (Cosier & Glazer, 1986) operating at 100.0 (1) K.
Geometry. All e.s.d.'s (except the e.s.d. in the dihedral angle between two l.s. planes) are estimated using the full covariance matrix. The cell e.s.d.'s are taken into account individually in the estimation of e.s.d.'s in distances, angles and torsion angles; correlations between e.s.d.'s in cell parameters are only used when they are defined by crystal symmetry. An approximate (isotropic) treatment of cell e.s.d.'s is used for estimating e.s.d.'s involving l.s. planes.
Refinement. Refinement of *F*^2^ against ALL reflections. The weighted *R*-factor *wR* and goodness of fit *S* are based on *F*^2^, conventional *R*-factors *R* are based on *F*, with *F* set to zero for negative *F*^2^. The threshold expression of *F*^2^ > σ(*F*^2^) is used only for calculating *R*-factors(gt) *etc*. and is not relevant to the choice of reflections for refinement. *R*-factors based on *F*^2^ are statistically about twice as large as those based on *F*, and *R*- factors based on ALL data will be even larger.

### Fractional atomic coordinates and isotropic or equivalent isotropic displacement parameters (Å^2^)

**Table:**

	*x*	*y*	*z*	*U*_iso_*/*U*_eq_	
F1	0.17366 (10)	0.87097 (10)	−0.57879 (6)	0.03431 (19)	
F2	0.40523 (9)	0.37977 (9)	0.20352 (6)	0.03302 (19)	
F3	0.15771 (9)	0.45771 (9)	0.49909 (6)	0.03172 (19)	
F4	0.54429 (8)	0.99409 (9)	0.24888 (6)	0.02763 (17)	
O1	−0.18938 (9)	0.62125 (9)	0.08627 (7)	0.02127 (17)	
O2	−0.12638 (9)	0.79463 (9)	0.21502 (7)	0.02271 (17)	
C1	0.09479 (14)	0.69493 (13)	−0.29224 (10)	0.0227 (2)	
H1A	0.0711	0.6060	−0.2430	0.027*	
C2	0.11646 (14)	0.71665 (14)	−0.40139 (10)	0.0261 (2)	
H2A	0.1074	0.6437	−0.4259	0.031*	
C3	0.15174 (13)	0.84918 (14)	−0.47200 (9)	0.0240 (2)	
C4	0.16684 (13)	0.96077 (13)	−0.43948 (9)	0.0221 (2)	
H4A	0.1912	1.0490	−0.4895	0.027*	
C5	0.14462 (12)	0.93777 (12)	−0.32982 (9)	0.0187 (2)	
H5A	0.1542	1.0113	−0.3061	0.022*	
C6	0.10798 (12)	0.80452 (11)	−0.25527 (8)	0.01738 (19)	
C7	0.08139 (12)	0.78028 (11)	−0.13880 (9)	0.01654 (19)	
C8	0.17578 (12)	0.83272 (11)	−0.08525 (8)	0.01669 (19)	
H8A	0.2581	0.8809	−0.1224	0.020*	
C9	0.14842 (11)	0.81386 (11)	0.02356 (8)	0.01554 (18)	
C10	0.02237 (11)	0.74476 (11)	0.07864 (8)	0.01598 (19)	
C11	−0.07150 (11)	0.68975 (11)	0.02521 (9)	0.01676 (19)	
C12	−0.04140 (12)	0.70569 (11)	−0.08225 (9)	0.01702 (19)	
H12A	−0.1025	0.6670	−0.1165	0.020*	
C13	−0.01691 (11)	0.72807 (11)	0.19450 (9)	0.01693 (19)	
C14	0.07608 (12)	0.62893 (12)	0.28288 (9)	0.0188 (2)	
H14A	0.0601	0.6290	0.3524	0.023*	
C15	0.18306 (12)	0.53861 (11)	0.26616 (9)	0.0178 (2)	
H15A	0.2000	0.5474	0.1945	0.021*	
C16	0.27629 (12)	0.42826 (12)	0.34593 (9)	0.0182 (2)	
C17	0.26488 (13)	0.38889 (13)	0.45849 (9)	0.0218 (2)	
C18	0.35385 (14)	0.28272 (14)	0.53126 (10)	0.0256 (2)	
H18A	0.3412	0.2608	0.6054	0.031*	
C19	0.46305 (14)	0.20924 (14)	0.49095 (10)	0.0279 (3)	
H19A	0.5247	0.1377	0.5386	0.033*	
C20	0.48077 (14)	0.24180 (14)	0.38016 (10)	0.0275 (3)	
H20A	0.5533	0.1927	0.3528	0.033*	
C21	0.38792 (13)	0.34881 (13)	0.31183 (9)	0.0221 (2)	
C22	0.25400 (11)	0.86676 (11)	0.07971 (8)	0.01586 (19)	
C23	0.40626 (12)	0.82182 (12)	0.08352 (9)	0.0199 (2)	
H23A	0.4414	0.7629	0.0474	0.024*	
C24	0.50582 (12)	0.86360 (13)	0.14022 (9)	0.0224 (2)	
H24A	0.6066	0.8328	0.1435	0.027*	
C25	0.44924 (12)	0.95271 (12)	0.19155 (9)	0.0201 (2)	
C26	0.30110 (13)	1.00312 (12)	0.18763 (9)	0.0202 (2)	
H26A	0.2678	1.0650	0.2217	0.024*	
C27	0.20311 (12)	0.95877 (11)	0.13136 (9)	0.0182 (2)	
H27A	0.1026	0.9908	0.1281	0.022*	
C28	−0.28775 (13)	0.56257 (13)	0.03620 (10)	0.0231 (2)	
H28A	−0.3641	0.5157	0.0877	0.035*	
H28B	−0.2319	0.4955	0.0117	0.035*	
H28C	−0.3334	0.6371	−0.0245	0.035*	

### Atomic displacement parameters (Å^2^)

**Table:**

	*U*^11^	*U*^22^	*U*^33^	*U*^12^	*U*^13^	*U*^23^
F1	0.0361 (4)	0.0511 (5)	0.0166 (3)	−0.0008 (4)	0.0005 (3)	−0.0142 (3)
F2	0.0317 (4)	0.0454 (5)	0.0188 (3)	0.0145 (3)	−0.0004 (3)	−0.0119 (3)
F3	0.0370 (4)	0.0357 (4)	0.0187 (3)	0.0152 (3)	0.0003 (3)	−0.0097 (3)
F4	0.0223 (3)	0.0393 (4)	0.0230 (3)	−0.0097 (3)	−0.0061 (3)	−0.0114 (3)
O1	0.0178 (4)	0.0265 (4)	0.0193 (4)	−0.0074 (3)	0.0010 (3)	−0.0073 (3)
O2	0.0168 (4)	0.0280 (4)	0.0234 (4)	0.0035 (3)	−0.0005 (3)	−0.0108 (3)
C1	0.0253 (5)	0.0232 (5)	0.0213 (5)	−0.0032 (4)	−0.0009 (4)	−0.0099 (4)
C2	0.0286 (6)	0.0317 (6)	0.0230 (5)	−0.0022 (5)	−0.0014 (4)	−0.0160 (5)
C3	0.0204 (5)	0.0365 (6)	0.0152 (5)	0.0014 (4)	−0.0014 (4)	−0.0105 (4)
C4	0.0188 (5)	0.0264 (5)	0.0177 (5)	0.0002 (4)	−0.0017 (4)	−0.0046 (4)
C5	0.0164 (4)	0.0209 (5)	0.0180 (5)	−0.0001 (4)	−0.0020 (4)	−0.0064 (4)
C6	0.0151 (4)	0.0207 (5)	0.0164 (4)	−0.0002 (3)	−0.0016 (3)	−0.0070 (4)
C7	0.0160 (4)	0.0169 (4)	0.0166 (4)	0.0005 (3)	−0.0018 (3)	−0.0063 (4)
C8	0.0144 (4)	0.0188 (4)	0.0165 (4)	−0.0007 (3)	−0.0003 (3)	−0.0063 (4)
C9	0.0125 (4)	0.0172 (4)	0.0167 (4)	0.0013 (3)	−0.0018 (3)	−0.0063 (4)
C10	0.0141 (4)	0.0173 (4)	0.0156 (4)	0.0011 (3)	−0.0019 (3)	−0.0054 (4)
C11	0.0145 (4)	0.0170 (4)	0.0173 (4)	−0.0007 (3)	−0.0008 (3)	−0.0048 (4)
C12	0.0160 (4)	0.0179 (4)	0.0174 (4)	−0.0012 (3)	−0.0023 (3)	−0.0067 (4)
C13	0.0145 (4)	0.0187 (4)	0.0178 (5)	−0.0014 (3)	−0.0007 (3)	−0.0070 (4)
C14	0.0175 (5)	0.0228 (5)	0.0156 (4)	0.0006 (4)	−0.0010 (4)	−0.0070 (4)
C15	0.0167 (4)	0.0201 (5)	0.0157 (4)	−0.0007 (4)	−0.0011 (3)	−0.0056 (4)
C16	0.0161 (4)	0.0210 (5)	0.0169 (5)	0.0000 (4)	−0.0010 (4)	−0.0067 (4)
C17	0.0215 (5)	0.0239 (5)	0.0191 (5)	0.0035 (4)	−0.0005 (4)	−0.0081 (4)
C18	0.0260 (6)	0.0292 (6)	0.0180 (5)	0.0046 (4)	−0.0031 (4)	−0.0058 (4)
C19	0.0242 (6)	0.0306 (6)	0.0235 (6)	0.0067 (5)	−0.0042 (4)	−0.0053 (5)
C20	0.0220 (5)	0.0328 (6)	0.0239 (6)	0.0095 (5)	−0.0010 (4)	−0.0086 (5)
C21	0.0190 (5)	0.0283 (5)	0.0173 (5)	0.0034 (4)	−0.0003 (4)	−0.0079 (4)
C22	0.0130 (4)	0.0187 (4)	0.0149 (4)	−0.0010 (3)	−0.0015 (3)	−0.0050 (4)
C23	0.0145 (4)	0.0244 (5)	0.0213 (5)	0.0008 (4)	−0.0011 (4)	−0.0094 (4)
C24	0.0130 (4)	0.0305 (6)	0.0222 (5)	−0.0002 (4)	−0.0033 (4)	−0.0082 (4)
C25	0.0180 (5)	0.0254 (5)	0.0158 (4)	−0.0059 (4)	−0.0043 (4)	−0.0050 (4)
C26	0.0206 (5)	0.0220 (5)	0.0192 (5)	−0.0026 (4)	−0.0009 (4)	−0.0087 (4)
C27	0.0147 (4)	0.0203 (5)	0.0194 (5)	0.0008 (3)	−0.0024 (3)	−0.0072 (4)
C28	0.0171 (5)	0.0268 (5)	0.0276 (6)	−0.0058 (4)	−0.0004 (4)	−0.0117 (5)

### Geometric parameters (Å, º)

**Table:**

F1—C3	1.3602 (13)	C13—C14	1.4756 (15)
F2—C21	1.3585 (13)	C14—C15	1.3440 (15)
F3—C17	1.3519 (13)	C14—H14A	0.9300
F4—C25	1.3656 (13)	C15—C16	1.4621 (15)
O1—C11	1.3645 (13)	C15—H15A	0.9300
O1—C28	1.4274 (14)	C16—C17	1.3985 (15)
O2—C13	1.2249 (13)	C16—C21	1.4002 (15)
C1—C2	1.3908 (16)	C17—C18	1.3791 (16)
C1—C6	1.3981 (16)	C18—C19	1.3910 (17)
C1—H1A	0.9300	C18—H18A	0.9300
C2—C3	1.3751 (18)	C19—C20	1.3882 (17)
C2—H2A	0.9300	C19—H19A	0.9300
C3—C4	1.3823 (18)	C20—C21	1.3782 (16)
C4—C5	1.3951 (15)	C20—H20A	0.9300
C4—H4A	0.9300	C22—C27	1.3958 (15)
C5—C6	1.4003 (15)	C22—C23	1.4010 (14)
C5—H5A	0.9300	C23—C24	1.3897 (16)
C6—C7	1.4850 (15)	C23—H23A	0.9300
C7—C8	1.3939 (15)	C24—C25	1.3819 (17)
C7—C12	1.4034 (15)	C24—H24A	0.9300
C8—C9	1.3990 (14)	C25—C26	1.3805 (16)
C8—H8A	0.9300	C26—C27	1.3912 (15)
C9—C10	1.3996 (14)	C26—H26A	0.9300
C9—C22	1.4888 (15)	C27—H27A	0.9300
C10—C11	1.4064 (15)	C28—H28A	0.9600
C10—C13	1.5123 (14)	C28—H28B	0.9600
C11—C12	1.3921 (15)	C28—H28C	0.9600
C12—H12A	0.9300		
			
C11—O1—C28	117.58 (9)	C14—C15—H15A	115.8
C2—C1—C6	121.10 (11)	C16—C15—H15A	115.8
C2—C1—H1A	119.5	C17—C16—C21	113.73 (10)
C6—C1—H1A	119.5	C17—C16—C15	126.22 (10)
C3—C2—C1	118.01 (11)	C21—C16—C15	120.04 (10)
C3—C2—H2A	121.0	F3—C17—C18	117.61 (10)
C1—C2—H2A	121.0	F3—C17—C16	117.92 (10)
F1—C3—C2	118.31 (11)	C18—C17—C16	124.46 (10)
F1—C3—C4	118.56 (11)	C17—C18—C19	118.36 (11)
C2—C3—C4	123.13 (11)	C17—C18—H18A	120.8
C3—C4—C5	118.34 (11)	C19—C18—H18A	120.8
C3—C4—H4A	120.8	C20—C19—C18	120.55 (11)
C5—C4—H4A	120.8	C20—C19—H19A	119.7
C4—C5—C6	120.35 (11)	C18—C19—H19A	119.7
C4—C5—H5A	119.8	C21—C20—C19	118.19 (11)
C6—C5—H5A	119.8	C21—C20—H20A	120.9
C1—C6—C5	119.07 (10)	C19—C20—H20A	120.9
C1—C6—C7	120.38 (10)	F2—C21—C20	117.95 (10)
C5—C6—C7	120.55 (10)	F2—C21—C16	117.34 (10)
C8—C7—C12	119.51 (10)	C20—C21—C16	124.70 (11)
C8—C7—C6	120.74 (9)	C27—C22—C23	118.94 (10)
C12—C7—C6	119.75 (10)	C27—C22—C9	120.92 (9)
C7—C8—C9	120.89 (10)	C23—C22—C9	120.12 (9)
C7—C8—H8A	119.6	C24—C23—C22	121.32 (10)
C9—C8—H8A	119.6	C24—C23—H23A	119.3
C8—C9—C10	119.59 (10)	C22—C23—H23A	119.3
C8—C9—C22	119.71 (9)	C25—C24—C23	117.43 (10)
C10—C9—C22	120.71 (9)	C25—C24—H24A	121.3
C9—C10—C11	119.51 (9)	C23—C24—H24A	121.3
C9—C10—C13	121.81 (9)	F4—C25—C26	117.91 (10)
C11—C10—C13	118.68 (9)	F4—C25—C24	118.65 (10)
O1—C11—C12	124.20 (10)	C26—C25—C24	123.44 (11)
O1—C11—C10	115.25 (9)	C25—C26—C27	118.14 (10)
C12—C11—C10	120.55 (10)	C25—C26—H26A	120.9
C11—C12—C7	119.86 (10)	C27—C26—H26A	120.9
C11—C12—H12A	120.1	C26—C27—C22	120.70 (10)
C7—C12—H12A	120.1	C26—C27—H27A	119.7
O2—C13—C14	120.22 (10)	C22—C27—H27A	119.7
O2—C13—C10	120.30 (9)	O1—C28—H28A	109.5
C14—C13—C10	119.47 (9)	O1—C28—H28B	109.5
C15—C14—C13	122.15 (10)	H28A—C28—H28B	109.5
C15—C14—H14A	118.9	O1—C28—H28C	109.5
C13—C14—H14A	118.9	H28A—C28—H28C	109.5
C14—C15—C16	128.48 (10)	H28B—C28—H28C	109.5
			
C6—C1—C2—C3	0.19 (18)	C11—C10—C13—C14	109.02 (11)
C1—C2—C3—F1	179.74 (11)	O2—C13—C14—C15	169.89 (11)
C1—C2—C3—C4	0.16 (19)	C10—C13—C14—C15	−8.99 (16)
F1—C3—C4—C5	−179.84 (10)	C13—C14—C15—C16	−175.29 (11)
C2—C3—C4—C5	−0.25 (18)	C14—C15—C16—C17	4.5 (2)
C3—C4—C5—C6	0.00 (16)	C14—C15—C16—C21	−176.78 (12)
C2—C1—C6—C5	−0.43 (17)	C21—C16—C17—F3	−178.53 (11)
C2—C1—C6—C7	178.74 (10)	C15—C16—C17—F3	0.30 (18)
C4—C5—C6—C1	0.33 (16)	C21—C16—C17—C18	0.26 (18)
C4—C5—C6—C7	−178.83 (10)	C15—C16—C17—C18	179.09 (12)
C1—C6—C7—C8	137.19 (11)	F3—C17—C18—C19	178.96 (12)
C5—C6—C7—C8	−43.66 (14)	C16—C17—C18—C19	0.2 (2)
C1—C6—C7—C12	−43.81 (15)	C17—C18—C19—C20	−0.4 (2)
C5—C6—C7—C12	135.34 (11)	C18—C19—C20—C21	0.3 (2)
C12—C7—C8—C9	−0.99 (15)	C19—C20—C21—F2	−179.68 (12)
C6—C7—C8—C9	178.01 (9)	C19—C20—C21—C16	0.2 (2)
C7—C8—C9—C10	−1.73 (15)	C17—C16—C21—F2	179.42 (11)
C7—C8—C9—C22	177.95 (9)	C15—C16—C21—F2	0.51 (17)
C8—C9—C10—C11	2.77 (15)	C17—C16—C21—C20	−0.45 (18)
C22—C9—C10—C11	−176.91 (9)	C15—C16—C21—C20	−179.36 (12)
C8—C9—C10—C13	−176.84 (9)	C8—C9—C22—C27	125.20 (11)
C22—C9—C10—C13	3.48 (15)	C10—C9—C22—C27	−55.12 (14)
C28—O1—C11—C12	−0.06 (15)	C8—C9—C22—C23	−56.29 (14)
C28—O1—C11—C10	−179.63 (9)	C10—C9—C22—C23	123.38 (11)
C9—C10—C11—O1	178.46 (9)	C27—C22—C23—C24	1.97 (16)
C13—C10—C11—O1	−1.92 (14)	C9—C22—C23—C24	−176.57 (10)
C9—C10—C11—C12	−1.12 (15)	C22—C23—C24—C25	−0.82 (17)
C13—C10—C11—C12	178.50 (9)	C23—C24—C25—F4	179.25 (10)
O1—C11—C12—C7	178.86 (10)	C23—C24—C25—C26	−1.08 (18)
C10—C11—C12—C7	−1.60 (15)	F4—C25—C26—C27	−178.59 (10)
C8—C7—C12—C11	2.64 (15)	C24—C25—C26—C27	1.74 (17)
C6—C7—C12—C11	−176.37 (9)	C25—C26—C27—C22	−0.51 (16)
C9—C10—C13—O2	109.75 (12)	C23—C22—C27—C26	−1.27 (16)
C11—C10—C13—O2	−69.86 (14)	C9—C22—C27—C26	177.25 (10)
C9—C10—C13—C14	−71.37 (14)		

### Hydrogen-bond geometry (Å, º)

*Cg*1 and *Cg*2 are the centroids of the C1–C6 and C7–C12 rings, respectively.

**Table:**

*D*—H···*A*	*D*—H	H···*A*	*D*···*A*	*D*—H···*A*
C2—H2*A*···F3^i^	0.93	2.46	3.3655 (18)	164
C8—H8*A*···F4^ii^	0.93	2.45	3.3726 (13)	170
C24—H24*A*···O2^iii^	0.93	2.57	3.4371 (14)	155
C20—H20*A*···*Cg*1^iv^	0.93	2.83	3.5082 (14)	130
C27—H27*A*···*Cg*2^v^	0.93	2.68	3.4068 (12)	136
C28—H28*B*···*Cg*2^vi^	0.96	2.90	3.7990 (15)	157

Symmetry codes: (i) *x*, *y*, *z*−1; (ii) −*x*+1, −*y*+2, −*z*; (iii) *x*+1, *y*, *z*; (iv) −*x*+1, −*y*+1, −*z*; (v) −*x*, −*y*+2, −*z*; (vi) −*x*, −*y*+1, −*z*.
